# Long Non-Coding RNA LOC113219358 Regulates Immune Responses in *Apis mellifera* Through Protein Interactions

**DOI:** 10.3390/ijms26020676

**Published:** 2025-01-15

**Authors:** Minjie Huang, Xiaodong Tan, Shuyuan Yang, Zhenzhen Zhou, Deqian Wang, Jie Dong

**Affiliations:** Institute of Animal Husbandry and Veterinary Science, Zhejiang Academy of Agricultural Sciences, Hangzhou 310021, China

**Keywords:** honeybee, *Apis mellifera*, lncRNA, immune regulation

## Abstract

Long non-coding RNAs (lncRNAs) are emerging as critical regulators in honeybee physiology, influencing development, behavior, and stress responses. This study investigates the role of lncRNA LOC113219358 in the immune response and neurophysiological regulation of *Apis mellifera* brains. Using RNA interference (RNAi) and RNA sequencing (RNA-seq), we demonstrate that silencing lncLOC113219358 significantly alters the expression of 162 mRNA transcripts, including genes associated with detoxification, energy metabolism, and neuronal signaling. Functional enrichment analysis revealed involvement in neuropeptide signaling, ATP synthesis, and oxidative phosphorylation pathways. Acetylcholinesterase (AChE), Glutathione-S-transferase (GST) and cytochrome P450 (CYP450) activities were significantly downregulated with 48 h of RNAi treatment. Additionally, RNA pull-down assays identified 113 proteins interacting with lncLOC113219358, including ATP synthase subunits, heat shock proteins, and major royal jelly proteins, suggesting its role in cellular stress responses and neural activity modulation. These findings provide mechanistic insights into how lncLOC113219358 mediates honeybee responses to environmental stressors, contributing to our understanding of lncRNA-regulated neural and immune functions in pollinators.

## 1. Introduction

Honeybees play a critical role in the pollination of numerous plant species, making substantial contributions to biodiversity conservation, agricultural productivity, and ecosystem stability [1,2]. As principal pollinators, honeybees facilitate the reproductive success of approximately 75% of flowering plants and the global crop production [3]. This essential pollination service is crucial for the production of fruits, vegetables, nuts, and seeds, which constitute fundamental components of human nutrition and various industrial applications [4]. Despite their importance, honeybee populations are experiencing significant declines globally, driven by a complex interplay of factors including habitat loss, pesticide exposure, climate change, and diseases [5,6]. These factors not only reduce the number of bees but also impair their ability to effectively pollinate plants. Additionally, the widespread use of pesticides, particularly neonicotinoids, has been demonstrated to exert detrimental effects on bee health and behavior, further exacerbating population declines [7,8]. Furthermore, the sublethal impacts of these pesticides exhibit synergistic interactions with concurrent environmental stressors, amplifying their deleterious effect on bee populations [9]. This convergence of chemical stressors with other environmental pressures has created a critical threat to bee survival, necessitating urgent conservation interventions.

Long non-coding RNAs (lncRNAs) are a class of non-protein-coding RNA molecules, which have been identified across numerous species [10,11]. Nevertheless, lncRNAs have emerged as key regulatory molecules with diverse functions in gene expression and cellular processes by interacting with the proteins and nucleic acids that regulate gene expression in the nucleus and cytoplasm. They are increasingly recognized for their role in honeybee development, behavior regulation, and stress responses [12,13,14]. For instance, the overexpression of lncRNA COV1 in the ovary of worker bees showed the consistent expression peak as autophagic cell death, which regulated the autophagic cell death of the ovary during the embryonic stage [15]. Studies on the responses of honeybee workers to *Ascospheara apis* or *Nosema ceranae* infection revealed the involvement of lncRNAs in mediating host–pathogen interactions [16,17]. Notably, Sawata et al. have reported a unique spatially- and temporary-regulated/sex-specific expression of non-coding RNA, Nb-1, which is involved in the honeybee lifecycle [18]. Consequently, these findings suggest that lncRNAs play a diverse array of regulatory roles in honeybees.

Previous studies have elucidated several specific functions of lncRNAs in the honeybee brain, including olfactory learning [19], dance behavior [20], responses to microsporidian infestation [21], and pesticide exposure [22]. In our preliminary study, we identified the lncLOC113219358 as a differentially expressed lncRNA in the honeybee (*Apis mellifera*) brain, demonstrating significant upregulation on both the 5th and 10th days following exposure to a sublethal dose of the neonicotinoid insecticide dinotefuran [22]. The full-length sequence of lncLOC113219358 is 471 bases and potentially targets four genes in trans, including general odorant-binding protein 71 and smoothelin-like protein 1. Moreover, a fluorescence in situ hybridization experiment revealed that lncLOC113219358 is predominantly localized to the cytoplasm of neuronal cells, suggesting a potential role in interacting with RNA-binding proteins [23]. Thus, the precise mechanisms underlying its function remain to be elucidated. In the present study, we explored the potential biological functions of lncLOC113219358 in the honeybee brain using RNA interference (RNAi) and RNA sequencing (RNA-seq). Additionally, we conducted RNA pull-down assays to identify and characterize lncLOC113219358-interacting proteins, thereby facilitating the prediction of their functional roles. This research lays a groundwork for elucidating the mechanisms by which long non-coding RNAs (lncRNAs) mediate honeybee responses to insecticide exposure.

## 2. Results

### 2.1. Results of dsRNA Synthesis and Interference Efficiency

**Figure 1:**
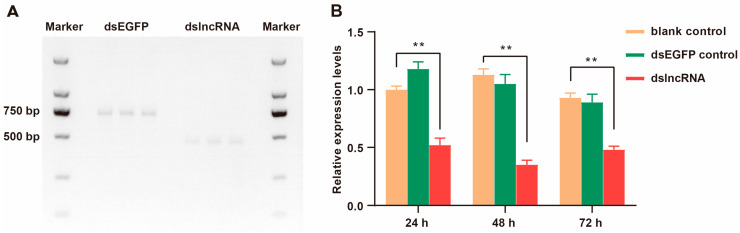
Synthesis and interference efficiency detection of dsRNA lncloc13129358. (**A**) Image of dsRNAs on agarose gel. (**B**) Efficiency of RNA interference with dslncLOC113219358 in *Apis mellifera ligustica* workers over time. Asterisks denote significant differences: ** *p* < 0.01.

Through the synthesis of dsRNA in vitro, as shown in Figure 1A, the dsRNA fragment bands were clear, and the sizes were consistent with expectations. The fragment of lncLOC113219358 was 471 bp, and the fragment of EGFP was 717 bp. The results of qRT-PCR showed that dslncLOC113219358 could play a role in the honeybee at 24 h, 48 h and 72 h after feeding, and the relative expression of lncLOC113219358 in the dsLOC feeding group was significantly lower than that in the dsEGFP feeding group (Figure 1B). The optimum interference time point after feeding was 48 h, and the interference time was selected for the subsequent experiment.

### 2.2. Effects of Silencing lncLOC113219358 on Gene Expression and Enzymatic Activities in the Honeybee

**Figure 2:**
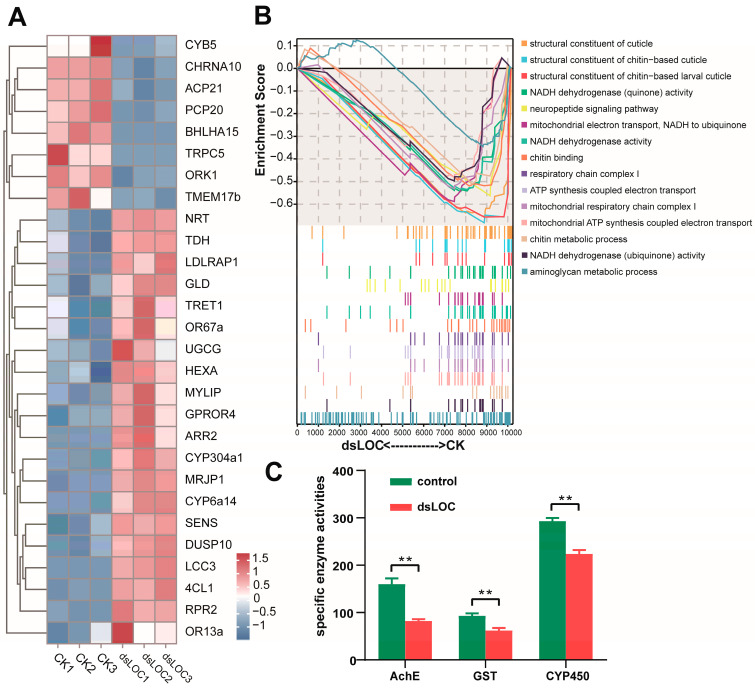
(**A**) Hierarchical clustering of differentially expressed genes. Red indicates relatively high expression, and blue indicates relatively low expression. (**B**) Gene Ontology–gene set enrichment analysis of mRNA expression in the honey bee brain. The vertical axis represents the corresponding running Enrichment Score (ES), with the peak value in the line graph indicating the ES of the gene set. ES > 0 indicates higher expression in the dsLOC group, and ES < 0 indicates higher expression in the CK group. The horizontal axis represents genes ranked in an ordered dataset. (**C**). Effect of lncLOC113219358 interference on enzyme activities of AChE, GST, and CYP450. Asterisks denote significant differences: ** *p* < 0.01.

After 48 h of RNAi treatment, compared with the control group (CK), 80 mRNA transcripts were upregulated, whereas 82 mRNA transcripts were downregulated in the dsRNA-treated group (dsLOC). Cytochrome P450 family proteins (CYP6a14 and CYP304a1) and glucose dehydrogenase (GLD), facilitated trehalose transporter (TRET1), neurotactin (NRT), and odorant receptors (OR67a and OR13a) were significantly upregulated in the dsRNA-treated group (Figure 2A). Meanwhile, short transient receptor potential channel 5-like (TRPC5), transmembrane protein 17B-like (TMEM17b), open rectifier potassium channel protein 1 isoform X1 (ORK1), and neuronal acetylcholine receptor subunit alpha-10 (CHRNA10) exhibited low expression in the dsRNA-treated group compared with the CK group. Gene set enrichment analysis indicated that the dsRNA-treated group was significantly associated with the neuropeptide signaling pathway, NADH dehydrogenase activity, respiratory chain complex I, and ATP synthesis-coupled electron transportation (Figure 2B). After 48 h of RNAi treatment, acetylcholinesterase (AChE), Glutathione-S-transferase (GST), and cytochrome P450 (CYP450) activities were significantly downregulated in the dsLOC honeybees compared with the control bees (*p* < 0.01) (Figure 2C).

### 2.3. Analysis of lncLOC113219358 Coding Potential

LncRNA can be translated to produce bioactive peptides via the presence of small open reading frames (sORFs) [24]. To investigate the coding potential, lncLOC113219358 was initially identified by the CPC2, CNCI, and FEELnc. As shown in Supplementary Table S2, lncLOC113219358 had no potential to encode proteins, which indicates that lncLOC113219358 may play a role in binding protein.

### 2.4. Proteomics Analysis Reveals Binding Proteins and Potential Roles

The main aim of this study was to identify the proteins that bound to lncLOC113219358 and their potential roles in the honeybee brain. To accomplish this objective, the proteins that were pulled down from lncLOC113219358 were subjected to LC-MS/MS analysis followed by bioinformatics analysis. A total of 4725 spectra were generated from the experiment. Based on the Mascot search results, 607 spectra matched known spectra, 559 matched peptides, and 113 matched proteins, respectively. More than 66.4% of the proteins included at least two peptides (Supplementary Table S3).

**Figure 3:**
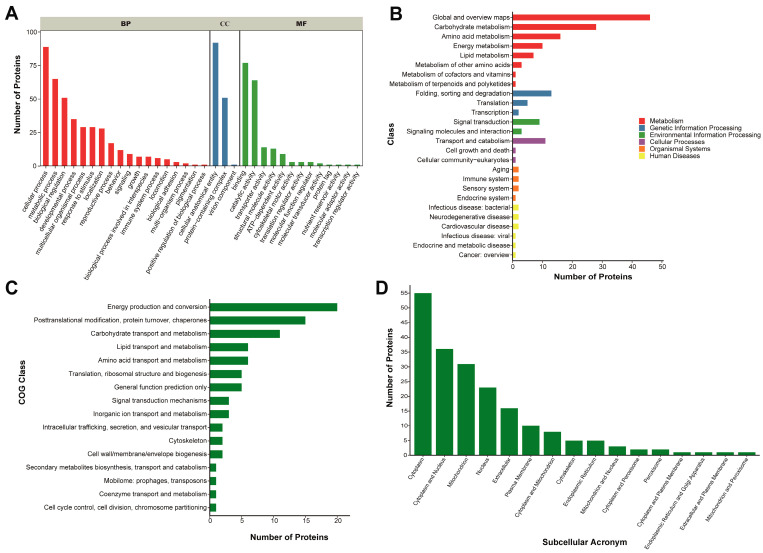
Functional annotation results of the lncLOC113219358 binding proteins. (**A**) GO function classification of the binding proteins. Legend: BP = Biological Process, CC = Cellular Component, MF = Molecular Function. (**B**) Statistic diagram of KEGG pathways annotation results. (**C**) COG annotation analyses of the binding proteins. (**D**) Subcellular localization prediction results of the binding proteins.

To explore the putative functions of the lncLOC113219358 and the binding proteins, GO terms and KEGG pathway analyses were performed. The results demonstrated that the binding proteins were annotated to biological process-associated terms such as cellular process, metabolic process, behavior, growth, and immune system process (Figure 3A), and molecular function-related terms such as binding, catalytic activity, and transporter activity. The binding proteins were annotated with the carbohydrate metabolism, amino acid metabolism, energy metabolism, signal transduction, and transport and catabolism (Figure 3B). As shown in Figure 3C, in addition to energy production and conversion-related proteins, the largest number is posttranslational modification, protein turnover, and chaperone-associated proteins in the cluster analysis of orthologous proteins of the genome, followed by carbohydrate transportation and metabolism and amino acid transportation and metabolism (Figure 3C). Subcellular localization information of proteins is important for biological activities, which contributes to a better understanding of the role of lncLOC113219358 in the honeybee brain. The annotation results show that the proteins bound to lncLOC113219358 were mainly located in the cytoplasm, cytoplasm and nucleus, and mitochondria and nucleus (Figure 3D).

## 3. Discussion

Long non-coding RNAs have emerged as pivotal regulators in various biological processes, exerting their functions through multiple mechanisms such as chromatin modification, transcriptional regulation, and post-transcriptional processing [25]. In this study, RNAi, RNA-seq and RNA pull-down were performed to identify the potential role of lncLOC113219358 in the honeybee brain. Bioinformatic analyses revealed that the reduction in lncLOC113219358 expression following dsRNA interference suggested its involvement in these intricate regulatory pathways, potentially influencing the expression of genes essential for honeybee physiological functions. The RNA pull-down results demonstrated that the bound proteins were annotated with the carbohydrate metabolism, amino acid metabolism, energy metabolism, signal transduction, and transport and catabolism.

The transcriptomic analysis following 48 h of dsRNA treatment targeting lncLOC113219358 (dsLOC) revealed substantial alterations in mRNA expression profiles compared to the control group (CK), underscoring the regulatory roles of this long non-coding RNA (lncRNA) in honeybee biology. A total of 80 mRNA transcripts were upregulated, while 82 mRNA transcripts were downregulated in the dsLOC-treated honeybees, indicating a broad impact on gene expression. Among the upregulated genes, several genes play crucial roles in honeybee physiology and adaptation. Cytochrome P450 family proteins, including CYP6a14 and CYP304a1, are known for their involvement in detoxification processes, particularly in metabolizing xenobiotics such as pesticides [26]. The upregulation of these enzymes suggests a potential response to environmental stressors or altered metabolic demands induced by lncRNA interference. Glucose dehydrogenase (GLD) and facilitated trehalose transporter (TRET1), upregulated in the dsLOC-treated group, are essential for carbohydrate metabolism and energy homeostasis in insects [27,28], and possibly in insect reproduction [29] and immunity [30]. These changes may reflect adjustments in energy metabolism pathways to compensate for altered regulatory signals caused by lncRNA reduction. Notably, Neurotactin (NRT), a cell adhesion molecule involved in neuronal development and synaptic plasticity [31], and odorant receptors (OR67a and OR13a), critical for olfactory perception and behavioral responses, were also found to be significantly upregulated. These findings suggest that lncLOC113219358 may influence neural development and sensory processes in honeybees, potentially affecting their learning, memory, and foraging behaviors [32]. Conversely, the downregulation of certain transcripts, such as short transient receptor potential channel 5-like (TRPC5), transmembrane protein 17B-like (TMEM17b), and neuronal acetylcholine receptor subunit alpha-10 (CHRNA10) in the dsLOC-treated group, indicates potential disruptions in ion channel activities and neurotransmitter signaling pathways [33,34,35]. These changes may affect neuronal excitability and synaptic transmission, further implicating lncLOC113219358 in modulating neural function and behavior in honeybees. ORK1, an open rectifier potassium channel protein, regulates potassium ion flux across cell membranes, crucial for maintaining electrical gradients and cellular excitability [36]. Reduced ORK1 expression may impact neuronal function and rhythmic activities essential for honeybee behavior. Gene set enrichment analysis (GSEA) further elucidated the biological pathways associated with the dsRNA-treated group, revealing significant enrichment in several key pathways. For instance, the neuropeptide signaling pathway is essential for modulating neuronal functions, behavior, and physiological responses in insects [37]; the respiratory chain complex I is involved in energy metabolism by catalyzing the transfer of electrons from NADH to ubiquinone, facilitating ATP synthesis [38], which is essential for cellular energy production and maintaining metabolic activities in honeybees [39]. These enrichments indicate that the reduction in lncLOC113219358 may impact energy metabolism, neuronal signaling, and cellular redox balance in honeybees. The observed changes in gene expression and pathway enrichments suggest a coordinated response to maintain cellular homeostasis and functional integrity under lncRNA-mediated regulatory influences.

Previous studies have shown that the enzymatic activities of acetylcholinesterase (AChE), Glutathione-S-transferase (GST), and cytochrome P450 (CYP450) play crucial roles in honeybee metabolism, detoxification, and response to pesticides [40,41,42]. The downregulation of AChE activity in dsLOC-treated honeybees suggests potential disruptions in cholinergic neurotransmission, affecting neuromuscular coordination and behavioral responses [43]. The reduction in GST and CYP450 activity following dsLOC treatment indicates a potential decrease in detoxification capacity, rendering honeybees more vulnerable to environmental toxins [44]. These enzymatic changes may reflect broader disruptions in metabolic pathways and stress responses induced by lncRNA interference, highlighting the interconnectedness of regulatory networks in bee biology.

LncRNAs were initially considered as having no coding capability [45]. However, recent studies have revealed that some lncRNAs may possess coding potential and could produce bioactive peptides through the presence of small open reading frames (sORFs) [24,46]. According to the results presented in coding potential, lncLOC113219358 is likely a non-coding RNA. The absence of protein-coding potential implies that lncLOC113219358 functions primarily through RNA-based mechanisms rather than through direct translation into proteins. Instead of acting as a template for protein synthesis, lncLOC113219358 might exert its biological effects through interactions with other biomolecules, such as proteins or other RNAs, thereby modulating their activities or stability [47].

Understanding the molecular interactions involving lncLOC113219358 could provide insights into its functional significance in honeybee physiology and adaptation. Consequently, we performed a pull-down of proteins interacting with lncLOC113219358, which assists in the exploration of the potential role of lncLOC113219358 in the honeybee brain. RNA pull-down results showed that a variety of proteins binding to lncLOC113219358 included ATP synthase subunit, heat shock proteins (HSPs), and major royal jelly proteins (MRJPs). ATP synthase is a key enzyme complex in oxidative phosphorylation, responsible for ATP production in mitochondria [48]. Honeybee workers exhibit exceptionally high metabolic rates during foraging flights, where their wing muscles demand substantial ATP [49]. ATP synthase subunits, as components of the mitochondrial electron transport chain, are directly involved in maintaining the cellular redox balance. The overproduction of reactive oxygen species (ROS) under stress can damage ATP synthase, reducing ATP production and compromising cellular functions [50]. In the context of pesticide exposure, for instance, studies suggest that neonicotinoids impair mitochondrial activity by targeting components of oxidative phosphorylation, including ATP synthase, exacerbating energy deficits and neurophysiological disruptions [51,52,53]. Our findings suggested that lncRNA LOC113219358 might interact with ATP synthase subunits in the honeybee brain, possibly influencing mitochondrial function and energy metabolism. HSPs are highly conserved molecular chaperones that play a pivotal role in cellular stress responses by maintaining protein homeostasis [54]. Exposure to sublethal doses of pesticides has been shown to upregulate HSP expression in honeybee brains, particularly HSP90 [55]. This response likely reflects an attempt to counteract pesticide-induced oxidative stress and neuronal damage, preserving cognitive functions, such as learning and memory, critical for navigation and foraging. However, chronic exposure to pesticides may overwhelm the protective capacity of HSPs, leading to cumulative damage and colony declines [56]. Our result suggested that MRJP1 and MRJP3 might interact with lncLOC113219358, which is a kind of nutrient-rich secretion produced by worker bees [57]. Studies have shown that in addition to its nutritional role, MJRP1 and MJRP3 provide protection against bacterial and fungal pathogens during larval development [58,59]. However, further RNA-Binding Protein Immunoprecipitation (RIP) investigation is needed to elucidate the precise molecular mechanisms underlying lncRNA and those protein interactions. Subcellular localization plays a crucial role in determining the function and regulation of proteins within cells [60]. In honeybee brains, cytoplasmic proteins involved in neurotransmitter synthesis and synaptic transmission contribute to neural signaling and information processing [61]. The presence of lncLOC113219358-associated proteins in the cytoplasm suggests potential roles in regulating these processes, influencing neuronal activity and behavior.

## 4. Materials and Methods

### 4.1. Honeybee Rearing

The honeybee colonies (*Apis mellifera*) were raised in the apiary at the Zhejiang Academy of Agricultural Sciences (Hangzhou, China). Newly emerged workers were collected from the frames within 24 h of eclosion and placed in a dark climate-controlled incubator (34 °C ± 1 °C, relative humidity 60% ± 10%) fed with adequate 50% (*w*/*v*) sucrose solution ad libitum.

### 4.2. dsRNA Synthesis, and RNAi Treatments

The synthesized LOC113219358 (LOC) gene was cloned into the *EcoRV* site of the pUC57 plasmid (pUC57-LOC) and confirmed by DNA sequencing. The pUC19-EGFP plasmid was donated by Dr. Hongchao Sun from Zhejiang Academy of Agricultural Sciences as a control. Standard PCR was performed using plasmids as templates for dsRNA synthesis with specific primers fused with the T7 5′- tail sequence (Supplementary Table S1) according to the manufacturer’s instructions of T7 RNAi Transcription Kit (Vazyme, Nanjing, China). The synthesized dsRNA-LOC (dsLOC) and dsRNA-EGFP (dsEGFP) products were purified by RNA clean beads. Then, dsRNA was checked for purity and integrity with a NanoPhotometer^®^ spectrophotometer (IMPLEN, Westlake Village, CA, USA) and 1% agarose gel. The dsRNA was diluted with nuclease-free water to a final concentration of 5 μg/μL and stored at −20 °C for the subsequent experiment.

Referring to the study of Nunes et al. [62], a non-invasive RNAi protocol was taken to avoid the impact of injection on the physiology and survival of honeybees. The bees were fed with a single 1 μL dose of a solution containing 5 μg of dsRNA (dsLOC or dsEGFP) using a micropipette, while 1 μL 50% sucrose–water solution was used as a blank control (dsCK). Bees from each treatment were collected at 24 h, 48 h, and 72 h after RNAi treatment, respectively. The bee samples were immediately preserved in liquid nitrogen and then stored at −80 °C for an optimal interference time test.

### 4.3. Sample Collection, RNA Extraction, Sequencing, and Enzyme-Linked Immunosorbent Assay

In the previous study, the optimal interference time (48 h) for dsRNA has been determined, and the results were shown in Figure 1B. The five-day bees were treated with 1 μL of 5 μg/μL dsRNA-LOC solution for 48 h in the experimental group (dsLOC), and 1 μL of 50% sucrose: water in the control group (CK) with three replicates for each treatment. The bees were fed ad libitum access to diet. All collected bees were promptly frozen in liquid nitrogen, and then the brains were dissected under the microscope.

Total RNA was isolated from the ten pooled brains in each group with three replicates using TRIzol reagent (Invitrogen, Carlsbad, CA, USA) and checked for the purity and integrity. A total of 3 μg was used to generate a complementary library with NEBNext^®^ Ultra™ RNA Library Prep Kit for Illumina^®^ following the manufacturer’s recommendations. After cluster generation using the TruSeq PE Cluster Kit v3-cBot-HS (Illumina, San Diego, CA, USA) according to the manufacturer’s instructions, three dsRNA-treated libraries and three control libraries were sequenced on the Illumina HiSeq 6000 platform by Gene Denovo Biotechnology Co. (Guangzhou, China). Bioinformatic analysis was performed using Omicsmart, a dynamic real-time interactive online platform for data analysis (http://www.omicsmart.com (accessed on 15 December 2024)).

Ten pooled bees treated with dsLOC were quickly ground with liquid nitrogen to powder and added to 0.01 mol/L PBS buffer. The powder was dissolved in PBS buffer (0.01 mol/L) and centrifuged at 5000 rpm for 15 min. Then, the cytochrome P450 (CYP450), glutathione s-transferase (GST), and acetylcholinesterase (AChE) levels in the supernatants were measured with enzyme-linked immunosorbent assay (ELISA) kits (KAITAI-Bio, Hangzhou, China) according to the manufacturer’s recommendations. The optical absorbance of the solution at 490 nm was measured with a spectrophotometer (Infinite F50, Tecan, Switzerland).

### 4.4. Potential Coding Analysis and RNA Pull-Down

LncLOC113219358 was subjected to coding potential prediction by the sequence intrinsic features-based prediction tool Coding Potential Calculator 2 (CPC2, http://cpc2.cbi.pku.edu.cn (accessed on 15 December 2024)), Coding-Non-Coding-Index (CNCI, https://github.com/www-bioinfo-org/CNCI (accessed on 15 December 2024)) and FlExible Extraction of LncRNAs (FEELnc, https://github.com/tderrien/FEELnc (accessed on 15 December 2024)) for potential coding analysis.

The interaction between RNA and protein was examined using the Pierce™ Magnetic RNA-Protein Pull-Down Kit (Thermo Fisher Scientific, Waltham, MA, USA) following the instructions of the manufacturer. The synthesis of lncLOC113219358 sense and lncLOC113219358 antisense RNAs were carried out with MAXIscript^®^ Kit (Thermo Fisher Scientific, Waltham, MA, USA) in accordance with the manufacturer’s instructions, and incubated with the proteins from the honeybee brain. Primer sequences used for amplification of the template DNA are shown in Supplementary Table S1. The RNA-binding protein complexes were washed, eluted, and detected by silver staining. Finally, the collected proteins were used for the mass spectrometry (MS) analysis (5600-plus, AB Sciex, Foster City, CA, USA).

### 4.5. qPCR and Statistical Analysis

Total RNA was isolated from the cells using TRIzol reagent and reverse-transcribed into first-strand cDNA with the PrimeScript™RT Reagent Kit with gDNA Eraser (TAKARA, Dalian, China). Real-time quantitative PCR was performed on an ABI 7500 Real-Time PCR system (Applied Biosystems, Foster City, CA, USA) with specific primers (Supplementary Table S1) in the following conditions: 95 °C for 30 s, 40 cycles of 95 °C for 5 s and 60 °C for 34 s, and subsequent melting curve analysis. The differences in the relative expression levels of genes were analyzed using an independent samples t-test with SPSS 22.0 software (IBM, Armonk, NY, USA) using the 2^−ΔΔCt^ method [63] with three independent biological replicates. *p* < 0.05 was considered statistically significant.

## 5. Conclusions

In conclusion, this research underscores the potential of lncRNAs as targets for mitigating honeybee population decline caused by stress and insecticide exposure. This study provides novel insights into the functional role of long non-coding RNA LOC113219358 in regulating the immune and physiological responses of *Apis mellifera*. Through RNA interference, transcriptomic analyses, and proteomic investigations, we demonstrate that lncLOC113219358 modulates a wide array of molecular pathways, including energy metabolism, detoxification, neuronal signaling, and immune regulation. The RNAi-mediated suppression of lncLOC113219358 significantly altered the expression of key genes involved in carbohydrate metabolism, neurodevelopment, and xenobiotic detoxification, underscoring its regulatory importance in maintaining honeybee homeostasis under environmental stressors such as pesticide exposure.

## Data Availability

The data presented in this study are available on request from the corresponding author due to the pending publication.

## Supplementary Materials

The following supporting information can be downloaded at: https://www.mdpi.com/article/10.3390/ijms26020676/s1.
